# Influence of Russian Vapor-Baths on the Cholera

**Published:** 1849-01

**Authors:** 


*Influence of Russian Vapor-baths on the Cholera.*—Of all the means employed against cholera, one of them from which the most efficacy is derived, is the vapor-bath. In some cases it has produced the most advantageous results in Russia, where its use is more generally adopted than in our climate. In the report of the medical commission, sent to Petersburg in 1830, we see that in the hospital of the hemp merchants, which contains all the materials for vapor-baths, out of forty cholera patients submitted to that treatment, six only died. Dr. Minchowsky, chief physician to the establishment, having, at the request of Drs. Barry and Russell, heated and fitted up the baths with vapor, as in the case of receiving patients for the cholera, two servants belonging to the hospital were sent with a thermometer, for the purpose of measuring the degree of heat. In the space of three minutes, the thermometer mounted, in the most elevated part of the building, to 46° Reaumur's scale, and in seven minutes it rose, upon the bench where the patients were placed, to 58° 12. Dr. Minchowsky, when a patient was brought in suffering under a severe effect of frost, placed him in the bath extended on the bench, and, after rubbing him with divers substances, applied the vapor of water and vinegar, until the circulation was restored, or until all hope of saving life had vanished. A patient who was at the last extremity, after being three hours in the vapor-bath at the high temperature, was restored to life. One of the physicians belonging to the commission, gives an interesting description of the vapor-baths in Russia, and the sensations he experienced, when he tried the effects on his own person.—*Med. Times.*

